# Common misconceptions and myths about ovarian cancer causation: a national cross-sectional study from palestine

**DOI:** 10.1186/s12889-024-18437-6

**Published:** 2024-04-12

**Authors:** Mohamedraed Elshami, Inas Jaber, Mohammed Alser, Ibrahim Al-Slaibi, Hadeel Jabr, Sara Ubaiat, Aya Tuffaha, Salma Khader, Reem Khraishi, Zeina Abu Arafeh, Sondos Al-Madhoun, Aya Alqattaa, Areej Yaseen, Asmaa Abd El Hadi, Ola Barhoush, Maysun Hijazy, Tamara Eleyan, Amany Alser, Amal Abu Hziema, Amany Shatat, Falasteen Almakhtoob, Balqees Mohamad, Walaa Farhat, Yasmeen Abuamra, Hanaa Mousa, Reem Adawi, Alaa Musallam, Shurouq I. Albarqi, Nasser Abu-El-Noor, Bettina Bottcher

**Affiliations:** 1 Division of Surgical Oncology, University Hospitals Cleveland Medical Center, 11100 Euclid Avenue, Lakeside 7100, 44106 Cleveland, OH USA; 2 Ministry of Health, Gaza, Palestine; 3 Faculty of Medicine, Al-Quds University, Jerusalem, Palestine; 4 United Nations Relief and Works Agency for Palestine Refugees (UNRWA), Gaza, Palestine; 5 Almakassed Hospital, Jerusalem, Palestine; 6 Faculty of Medicine, Al-Quds University, Bethlehem, Palestine; 7 Al-Watani Hospital, Nablus, Palestine; 8 Faculty of Medicine, An-Najah National University, Nablus, Palestine; 9 Al-shiffa Hospital, Gaza, Palestine; 10 Faculty of Medicine, Islamic University of Gaza, Gaza, Palestine; 11 Faculty of Medicine, Palestine Polytechnic University, Hebron, Palestine; 12 Doctors Without Borders (Médecins Sans Frontières), Hebron, Palestine; 13 Faculty of Medicine, Al-Azhar University-Gaza, Jenin, Palestine; 14 Faculty of Medicine, Al-Azhar University-Gaza, Gaza, Palestine; 15 Al-Aqsa Hospital, Deir Albalah, Palestine; 16 Faculty of Pharmacy, Al-Azhar University of Gaza, Gaza, Palestine; 17 Faculty of Nursing, Islamic University of Gaza, Gaza, Palestine

**Keywords:** Ovarian cancer, Myths, Mythical causes, Beliefs, Behavioral changes, Palestine

## Abstract

**Background:**

Women’s inability to recognize ovarian cancer (OC) causation myths to be incorrect may lead to behavioral changes that could distract them from actual risk factors and impact their treatment decision making. This study examined Palestinian women’s recognition of OC mythical causes, and explored factors associated with good recognition.

**Methods:**

A national cross-sectional study was conducted. Adult Palestinian women were recruited from hospitals, primary healthcare facilities, and public areas in 11 governorates. The Cancer Awareness Measure-Mythical Causes Scale was modified and utilized for data collection. Awareness level was determined based on the number of myths around OC causation recognized to be incorrect: poor (0–4), fair (5–9), and good (10–13).

**Results:**

A total of 5618 participants agreed and completed the questionnaire out of 6095 approached (response rate = 92.1%), and 5411 questionnaires were included in the final analysis. The most recognized food-related myth was ‘drinking from plastic bottles’ (*n* = 1370, 25.3%) followed by ‘eating burnt food’ (*n* = 1298, 24.0%). The least recognized food-related myth was ‘eating food containing additives’ (*n* = 611, 11.3%). The most recognized food-unrelated myth was ‘having a physical trauma’ (*n* = 2899, 53.6%), whereas the least recognized was ‘using mobile phones’ (n = 1347, 24.9%). Only 273 participants (5.1%) had good awareness of OC causation myths as incorrect. Earning higher monthly incomes as well as visiting governmental healthcare facilities were associated with a decrease in the likelihood of exhibiting good awareness.

**Conclusion:**

The overall recognition of OC causation myths was low. Addressing mythical beliefs should be included in OC prevention strategies and public health interventions to improve women’s understanding of OC risk factors versus mythical causes.

**Supplementary Information:**

The online version contains supplementary material available at 10.1186/s12889-024-18437-6.

## Background

The GLOBOCAN 2020 report estimates a total of 313,959 new ovarian cancer (OC) cases and 207,252 deaths in 2020, with the disease being the eighth most common cancer in women globally, both in terms of incidence and mortality [1]. Despite the declining incidence and mortality rates for OC over the past decade, a substantial increase in incidence was observed among younger females [2]. In Palestine, 74 new cases of OC and 53 deaths were recorded in 2022 [3]. Importantly, OC is the seventh most common cancer among Palestinian women, with an incidence rate of 3.7 per 100,000 female population [4].

The high morbidity and mortality of OC worldwide is largely due to late presentation with the disease (stage III or stage IV) caused by the nonspecific initial OC symptoms that can also indicate various benign diseases [5, 6]. Early diagnosis results in effective management with a 5-year survival rate of up to 90% compared with 30% and less for advanced stages [7]. Early diagnosis can be facilitated by increasing the awareness of OC symptoms and risk factors, appropriate health-seeking behavior, and accessible healthcare [8].

Low- and middle-income countries are facing rising rates of OC cases and have lower survival rates than high-income countries [6, 8, 9]. Knowledge of OC symptoms and awareness levels regarding risk and protective factors among women in Palestine were found to be low [10, 11]. This might contribute to the late presentation with possible OC symptoms. Notably, there is a gap in the literature about the recognition of myths related to OC causation in the Palestinian community. Previous studies classified those mythical causes of OC into food-related (e.g., drinking from plastic bottles, eating burnt food) and food-unrelated (e.g., having a physical trauma, using aerosol containers, feeling stressed) [12–14]. The inability to recognize such myths to be incorrect may lead to behavioral changes that might draw away attention from well-established risk factors and may affect treatment decision making [12].

Therefore, this study aimed to (i) assess Palestinian women’s awareness of OC causation myths to be incorrect, (ii) compare recognition levels among women living in the Gaza Strip vs. the West Bank and Jerusalem (WBJ), and (iii) identify the factors associated with good awareness of OC causation myths.

## Materials and methods

### Study design and population

Our group previously demonstrated a low awareness of OC symptoms as well as risk and protective factors [10, 11]. This was a follow-up study to examine the awareness of myths surrounding OC causation among Palestinian women through a national survey in the WBJ and the Gaza Strip between July 2019 and March 2020. The study included governmental hospitals, primary healthcare centers, and public venues distributed across 11 governorates (seven in the WBJ and four in the Gaza Strip). The public venues incorporated in the study included different locations, including shopping centers, markets, parks, restaurants, mosques, churches, and transport networks. Palestinian women ≥ 18 years old who attended one of the designated data collection sites at the time of data collection were invited to participate. Women holding non-Palestinian nationality, women working or studying in a health-related field, visitors to oncology departments, as well as those unable to complete the questionnaire were all excluded.

### Sampling size calculation

In Palestine, there were 1,534,371 females who were 15 years of age or older in 2019 [15]. At a 95.0% confidence level, 5.0% type I error rate, and 1.0% absolute error, 900 participants were the minimum sample size needed to detect a 10% good overall awareness of OC causation myths.

### Sampling methods

Convenience sampling was used to recruit eligible women. The data collection sites were intentionally distributed across multiple locations in Palestine to enhance the representativeness of the study cohort and ensure broader inclusivity of the Palestinian community. This approach aimed to capture a more comprehensive and diverse sample as described in previous reporting [10, 11, 16–19].

### Questionnaire and data collection

Data were collected using a modified version of the Cancer Awareness Measure–Mythical Causes Scale (CAM-MYCS) [12]. The CAM-MYCS was translated from English to Arabic by two bilingual healthcare professionals and subsequently back-translated from Arabic to English by two other bilingual healthcare professionals. All of these healthcare professionals possessed relevant expertise in gynecologic oncology, public health, and survey design. To ensure the questionnaire’s content validity, five independent healthcare professionals and researchers evaluated its content. A pilot study (*n* = 128) was conducted to assess the clarity of the Arabic questionnaire. Notably, the questionnaires used in the pilot study were excluded from the final analysis. The questionnaire demonstrated acceptable internal consistency, with a Cronbach’s Alpha coefficient of 0.73.

The questionnaire consisted of two sections. The first section captured sociodemographic information, including variables such as age, highest level of education attained, employment status, monthly income, marital status, knowing someone with cancer, place of residence, presence of any chronic illnesses, and the site of data collection. The second section focused on evaluating participants’ ability to identify and recognize 13 myths related to OC causation as being incorrect. All but one of these myths were derived from the original CAM-MYCS. The item ‘eating burnt food’ was deemed important to be added [20].

For the current study, the Arabic questionnaire was adapted from the original CAM-MYCS to better suit the study purposes. The questions from the CAM-MYCS, which originally had response options of correct/incorrect/unsure, were converted into a 5-point Likert scale (1 = strongly disagree, 5 = strongly agree). This conversion was intended to minimize the possibility of providing random responses. Responses with ‘strongly disagree’ or ‘disagree’ were considered correct, while all other response options were regarded as incorrect.

The electronic tool ‘Kobo Toolbox’, which is an offline/online tool accessed by smart devices, was used by trained data collectors for data collection [21]. Eligible women had face-to-face interviews with data collectors who had been trained on using Kobo Toolbox, approaching participants, and facilitating questionnaire completion.

### Statistical analysis

Descriptive statistics were utilized to describe patient characteristics. Categorical variables were described using frequencies and percentages, while continuous variables, which exhibited a non-normal distribution, were described using the median and interquartile range (IQR).

To compare baseline characteristics between participants recruited from the Gaza Strip and those recruited from the WBJ, the Kruskal-Wallis test was utilized for continuous variables, and Pearson’s Chi-square test was utilized for categorical variables.

To account for the age-related risk of OC, the participants’ ages were grouped into two categories: 18 to 44 years and 45 years or older, with the latter considered as the at-risk group [22]. For monthly income, a cutoff of 1450 NIS (approximately $450) was selected to create two categories. This value was chosen because it represents the minimum wage in Palestine [23].

The examined myths around OC causation were categorized into two groups: food-related and food-unrelated. The recognition of each myth was described using frequencies and percentages. To compare the recognition of myths between participants from the Gaza Strip and those from the WBJ, Pearson’s Chi-Square test was employed. To examine the association between participant characteristics and the recognition of each myth as being incorrect, multivariable logistic regression analyses were utilized. The multivariable analyses adjusted for several covariates, including age-group, educational level, employment status, monthly income, marital status, place of residency, presence of a chronic disease, knowing someone with cancer, and site of data collection. This model was predetermined based on previous studies [10–12, 24, 25].

The awareness level regarding OC causation myths was assessed using a scoring system, which had been utilized in prior studies [10, 11, 16–19]. For each correctly identified myth, participants were given one point. The total score, ranging from 0 to 13, was calculated, and participants were then categorized into three awareness levels based on the number of myths correctly recognized as incorrect: poor (0 to 4 points), fair (5 to 9 points), and good awareness (10 to 13 points). To compare the ability to recognize myths around OC causation between participants from the Gaza Strip and those from the WBJ, Pearson’s Chi-Square test was employed. Additionally, a multivariable logistic regression analysis was conducted to investigate the association between participant characteristics and displaying good awareness. The same multivariable model mentioned earlier was used for this purpose. Furthermore, a sensitivity analysis was performed, where co-variates significantly associated with displaying good awareness of OC causation myths on univariable analysis were only included in the multivariable model.

Missing data were hypothesized to have been missed completely at random and thus, complete case analysis was utilized to handle them. Data were analyzed using Stata software version 17.0 (StataCorp, College Station, Texas, United States).

## Results

### Participant characteristics

From the 6095 potential participants approached, 5618 participants agreed and completed the questionnaire (response rate = 92.1%). A total of 5411 questionnaires were included in the final analysis (49 did not meet inclusion criteria and 158 had some missing values); 2278 from the Gaza Strip and 3133 from the WBJ. Participants living in the WBJ were older, earned higher monthly income, and had more frequent chronic diseases than those living in the Gaza Strip (Table 1).

**Table 1:** Characteristics of study participants

Characteristic	Total (*n*= 5411)	Gaza Strip (*n*= 2278)	WBJ (*n*= 3133)	p-value
**Age**, median [IQR]	32.0 [24.0,44.0]	30.0 [24.0,40.0]	34.0 [25.0,46.0]	<0.001
**Age group**, n (%)				
18 to 44	4151 (76.7)	1872 (82.2)	2279 (72.7)	<0.001
45 or older	1260 (23.3)	406 (17.8)	854 (27.3)	
**Menarche**, n (%)				
Early (≤ 10 years)	65 (1.2)	20 (0.9)	45 (1.4)	<0.001
Normal (11-15 years)	4658 (86.1)	1923 (84.4)	2735 (87.3)	
Late (≥ 16 years)	688 (12.7)	335 (14.7)	353 (11.3)	
**Educational level**, n (%)				
Secondary or below	3016 (55.7)	1330 (58.4)	1686 (53.8)	<0.001
Post-secondary	2395 (44.3)	948 (41.6)	1447 (46.2)	
**Occupation**, n (%)				
Unemployed/housewife	3671 (67.8)	1837 (80.6)	1834 (58.5)	<0.001
Employed	1095 (20.2)	254 (11.2)	841 (26.8)	
Retired	47 (0.9)	9 (0.4)	38 (1.2)	
Student	598 (11.1)	178 (7.8)	420 (13.4)	
**Monthly income ≥ 1450 NIS**, n (%)	3330 (61.5)	474 (20.8)	2856 (91.2)	<0.001
**Marital status**, n (%)				
Single	1248 (23.1)	374 (16.4)	874 (27.9)	<0.001
Married	3952 (73.0)	1836 (80.6)	2116 (67.5)	
Divorced/Widowed	211 (3.9)	68 (3.0)	143 (4.6)	
**Having a chronic disease**, n (%)	1097 (20.3)	350 (15.4)	747 (23.8)	<0.001
**Knowing someone with cancer**, n (%)	2746 (50.8)	1104 (48.5)	1642 (52.4)	0.004
**Site of data collection**, n (%)				
Public Spaces	1645 (30.4)	596 (26.2)	1049 (33.5)	<0.001
Hospitals	1735 (32.1)	650 (28.5)	1085 (34.6)	
Primary healthcare centers	2031 (37.5)	1032 (45.3)	999 (31.9)	

n= number of participants, IQR= interquartile range, WBJ= West Bank and Jerusalem, OC= ovarian cancer

### Recognition of OC causation myths to be incorrect

In general, OC myths unrelated to food were more commonly recognized as incorrect, than those related to food. The most recognized food-related myth to be incorrect was ‘drinking from plastic bottles’ (*n* = 1370, 25.3%) followed by ‘eating burnt food’ (*n* = 1298, 24.0%) (Table 2). The least recognized food-related myth to be incorrect was ‘eating food containing additives’ (*n* = 611, 11.3%). The most recognized food-unrelated myth around OC causation to be incorrect was ‘having a physical trauma’ (*n* = 2899, 53.6%), whereas the least recognized was ‘using mobile phones’ (n = 1347, 24.9%).

**Table 2:** Summary of the assessment of public beliefs in mythical causes of ovarian cancer

Myth	Total (*n*= 5411) n (%)	Gaza Strip (*n*= 2278) n (%)	WBJ (*n*= 3133) n (%)	p–value
Food-related				
Drinking from plastic bottles	1370 (25.3)	592 (26.0)	778 (24.8)	0.33
Eating burnt food (e.g., bread or barbeque)	1298 (24.0)	390 (17.1)	908 (29.0)	<0.001
Eating food containing artificial sweeteners (e.g., saccharine)	1013 (18.7)	429 (18.8)	584 (18.6)	0.86
Using microwave ovens	958 (17.7)	369 (16.2)	589 (18.8)	0.013
Eating genetically modified food (e.g., hybrid vegetables)	689 (12.7)	312 (13.7)	377 (12.0)	0.07
Eating food containing additives	611 (11.3)	240 (10.5)	371 (11.8)	0.13
Others				
Having a physical trauma	2899 (53.6)	1306 (57.3)	1593 (50.8)	<0.001
Using aerosol containers	2127 (39.3)	822 (36.1)	1305 (41.7)	<0.001
Using cleaning products	1758 (32.5)	752 (33.0)	1006 (32.1)	0.48
Feeling stressed	1562 (28.9)	668 (29.3)	894 (28.5)	0.53
Living near power lines	1386 (25.6)	742 (32.6)	644 (20.6)	<0.001
Exposure to electromagnetic frequencies (e.g., Wi-Fi and Radio/TV frequencies)	1365 (25.2)	585 (25.7)	780 (24.9)	0.51
Using mobile phones	1347 (24.9)	503 (22.1)	844 (26.9)	<0.001

n= number of participants. WBJ= West Bank and Jerusalem

### Good awareness of myths around OC causation and its associated factors

Only 273 participants (5.1%) displayed good recognition of OC myths to be incorrect (i.e., promptly recognized more than nine out of 13 OC myths) (Table 3). Participants from both the Gaza Strip and the WBJ had similar likelihoods to display good recognition for the listed OC myths. (5.4% vs. 4.7% respectively). Earning higher monthly income (≥ 1450 NIS) (OR = 0.54, 95% CI: 0.37–0.79) as well as visiting hospitals (OR = 0.65, 95% CI: 0.47–0.90) and primary healthcare centers (OR = 0.69, 95% CI: 0.51–0.94) were all associated with a decrease in the likelihood of recognizing myths around OC causation to be incorrect (Table 4). Consistent findings were also observed on sensitivity analysis that adjusted for participant characteristics that were associated with displaying good awareness of OC causation myths on univariable analysis (supplementary Table 1).

**Table 3:** Awareness level of mythical causes of ovarian cancer among study participants

Level	Total n (%)	Gaza Strip n (%)	WBJ n (%)	p–value
**Poor** (0–4 myths)	3783 (69.9)	1594 (70.0)	2189 (69.9)	0.46
**Fair** (5–9 myths)	1355 (25.0)	560 (24.6)	795 (25.4)	
**Good** (10–13 myths)	273 (5.1)	124 (5.4)	149 (4.7)	

n= number of participants, WBJ= West Bank and Jerusalem

**Table 4:** Bivariable and multivariable logistic regression analyzing factors associated with having good recognition of the mythical causes of ovarian cancer

Characteristic	Good recognition			
	Crude OR (95% CI)	p-value	AOR (95% CI)*	p-value
**Age group**				
18 to 44	Ref	Ref	Ref	Ref
45 or older	0.67 (0.49- 0.93)	0.015	0.98 (0.67- 1.41)	0.89
**Educational level**				
Secondary or below	Ref	Ref	Ref	Ref
Post-secondary	1.15 (0.90- 1.47)	0.25	1.09 (0.82- 1.43)	0.56
**Occupation**				
Housewife	Ref	Ref	Ref	Ref
Employed	0.91 (0.66- 1.27)	0.59	0.83 (0.57- 1.22)	0.35
Retired	0.44 (0.06- 3.23)	0.42	0.53 (0.07- 4.02)	0.54
Student	1.98 (1.44- 2.73)	<0.001	1.30 (0.84- 1.99)	0.24
**Monthly income**				
< 1450 NIS	Ref	Ref	Ref	Ref
≥ 1450 NIS	0.73 (0.57- 0.93)	0.011	0.54 (0.37- 0.79)	0.001
**Marital status**				
Single	Ref	Ref	Ref	Ref
Married	0.56 (0.43- 0.73)	<0.001	0.71 (0.50- 1.01)	0.06
Divorced/Widowed	0.36 (0.16- 0.83)	0.017	0.45 (0.19- 1.09)	0.08
**Residency**				
Gaza Strip	Ref	Ref	Ref	Ref
WBJ	0.87 (0.68- 1.11)	0.25	1.31 (0.89- 1.91)	0.17
**Having a chronic disease**				
No	Ref	Ref	Ref	Ref
Yes	0.56 (0.39- 0.81)	0.002	0.68 (0.45- 1.01)	0.06
**Knowing someone with cancer**				
No	Ref	Ref	Ref	Ref
Yes	1.02 (0.80- 1.30)	0.86	1.03 (0.80- 1.32)	0.81
**Site of data collection**				
Public Spaces	Ref	Ref	Ref	Ref
Hospitals	0.56 (0.41- 0.76)	<0.001	0.65 (0.47- 0.90)	0.009
Primary healthcare centers	0.64 (0.48- 0.85)	0.002	0.69 (0.51- 0.94)	0.018

OR= odds ratio, AOR= adjusted odds ratio, CI= confidence interval, WBJ= West Bank and Jerusalem

Note: The outcome was dichotomized, where displaying good awareness was considered as ‘yes’, whereas displaying poor or fair awareness was considered as ‘no’

* Adjusted for age-group, educational level, occupation, monthly income, marital status, residency, having a chronic disease, knowing someone with cancer, and site of data collection

### Association between participant characteristics and recognition of OC food-related myths

Married participants were less likely than single participants to recognize all OC food-related myths around OC causes to be incorrect except ‘eating burnt food’, where no associated difference was observed (supplementary Table 2). In addition, having a higher monthly income and visiting primary healthcare centers were associated with a decrease in the likelihood of recognizing half of the OC food-related myths as incorrect. In contrast, participants living in the WBJ were more likely than participants living in the Gaza Strip to recognize half of the OC food-related myths to be incorrect. Both, participants who completed or did not complete higher education (i.e., postsecondary education), had similar likelihoods to recognize all OC food-related myths to cause OC to be incorrect.

### Association between participant characteristics and recognition of OC myths unrelated to food

Participants recruited from primary healthcare centers were less likely than participants recruited from public spaces to recognize all OC myths not related to food to be incorrect except for ‘using cleaning products’, where no associated difference was observed (supplementary Table 3). In addition, participants who were 45 years or older were less likely than younger participants to recognize five out of seven OC myths not related to food as incorrect. Participants with postsecondary education were less likely to recognize ‘using cleaning products’ (OR = 0.85, 95% CI: 0.74–0.97) but more likely to recognize ‘using mobile phones’ (OR = 1.16, 95% CI: 1.00-1.33) than participants with lower education levels. Both, participants who completed or did not complete higher education, had similar likelihoods to recognize all other OC myths not related to food as incorrect.

## Discussion

This study showed that only 5.1% of the study participants had good abilities to recognize myths around OC causation to be incorrect (i.e., recognizing more than nine out of 13 OC myths). WBJ and the Gaza Strip participants demonstrated similar recognition of OC myths. The factors associated with lower abilities to recognize OC myths to be incorrect were earning higher monthly income and visiting governmental healthcare facilities. The most recognized food-related myth was ‘drinking from plastic bottles’, while the least was ‘eating food containing additives’. The most recognized myth unrelated to food was ‘having a physical trauma’, whereas the least recognized was ‘using mobile phones’.

### Recognition of OC myths as incorrect

OC mythical beliefs may raise anxiety levels and result in the divergence of energy to avoid OC actual causes [26]. In concordance with previous studies from other countries [12, 26–29], Palestinian women’s ability to recognize myths around OC causation to be incorrect was poor. Previous studies investigated Palestinian women’s awareness of actual OC symptoms as well as risk and protective factors and found it also to be poor [10, 11]. This study adds to the literature by reporting on the understanding of OC mythical causes among Palestinian women and the possible areas for improvement in future educational interventions. Low- and middle-income countries such as Palestine encounter various challenges in combating OC, including late presentation for both diagnosis and treatment [9]. The poor recognition of OC causation myths in this study coupled with the low awareness of OC symptoms and risk factors in previous studies [10, 11] encourage the implementation of interventions that promote public knowledge around OC, and engagement with healthcare systems in order to reduce stress and boost women’s confidence about their ability to take control and reduce their risks [26, 30].

Misconceptions can spread by sharing misinterpreted experiences as well as poor knowledge of OC risk factors, causes, treatment options and outcomes [31, 32]. Understanding how mythical beliefs influence health behaviors could help design more efficient educational interventions and strategies to prevent OC and promote public health [12]. Of note, social media may provide a good platform for women to engage in dialogues with experts, educate themselves, receive support from peers, and advocate for their care with the goal of boosting their health literacy and ultimately their health outcomes [33, 34].

### Association between participant characteristics and recognition of OC causation myths

Previous studies have shown that married women were more likely to display good awareness levels of OC symptoms, risk and protective factors [10, 11, 16, 35]. However, in this study, they had poor recognition of OC causation myths that were related to food. This is consistent with the literature, where it has been demonstrated that married women are vulnerable to such myths especially during periods of pregnancy, postpartum and even while breastfeeding [36–38]. The origin of these myths could be mainly driven by cultural beliefs and regular avoidance of specific behaviors related to diet that turns into a tradition that is shared among married women [36, 39].

Shahab and colleagues showed that lower educational levels were associated with poor recognition of cancer myths to be incorrect [26]. However, we found both participants who completed or did not complete higher education to have similar likelihoods to recognize all OC myths to be incorrect. This could be attributed to improved awareness due to the School Health Program, initiated by the Palestinian Ministry of Health, which provides students not only with medical services but also health education activities and workshops [4]. Furthermore, it was reported that 70.6% of the total population in Palestine used internet in 2022, and there has been a growing trend to utilize social media for educational purposes of different topics including those related to healthcare [40, 41]. All these factors may have reduced the gap in OC myth recognition between participants with different educational backgrounds.

Participants recruited from hospitals and primary healthcare centers were less likely to have good recognition of myths to be incorrect. A previous national study in Palestine showed that participants recruited from primary healthcare centers had lower likelihood of displaying good awareness of OC risk factors [10]. Possible explanations for this finding could be that women attending hospitals and primary healthcare centers do not use available educational resources, or they gain little additional knowledge from their interaction with healthcare professionals. Elshami and colleagues examined the barriers to early presentation with possible cancer symptoms and showed that women might worry about wasting the doctor’s time, feel embarrassed, and worry about what the doctor might find [32, 42]. Such feelings might impede potential improvements in health literacy that may result from visits to hospitals and primary healthcare centers and, thus, exacerbate the poor recognition of myths around OC causation [32, 42].

Whereas WBJ and the Gaza Strip both belong to the occupied Palestinian territories, no actual geographical connection exists between these two areas. Residents of the Gaza Strip need a special permit to travel to WBJ, which is only rarely granted [43]. Likewise, residents of the WBJ cannot not travel to the Gaza Strip with such a permit and they are equally unlikely to be granted permission to enter the Gaza Strip. Therefore, the residents of these two areas although they belong to one country, have very little actual opportunities for face-to-face communication or building relationships. This led to the assumption that it might be likely for the awareness of these two groups of Palestinian residents of myths around OC causation to differ significantly. However, this study found no significant difference in their ability to recognize myths around OC causation, pointing to the possibility of health information being accessed and gained from online and social media sources [40, 41].

### Future directions

The finding of this study underline the fundamental necessity to establish sustainable educational programs that aim to raise public awareness of OC causation myths and risk factors. Targeting common myths and misconceptions around OC and explaining why they are false or misleading through brochures, videos, podcasts, or social media campaigns is recommended. Furthermore, there is a need to measure the effectiveness of such interventions by assessing the changes in knowledge, attitudes, and behaviors of women before and after the intervention. This might achieve an increase in awareness levels of OC and may boost women’s confidence and, thus, encourage them to approach healthcare professionals and discuss their symptoms as soon as they recognize them.

### Limitations

The present study is subject to certain limitations. Notably, the use of convenience sampling for recruitment of participants, may have impacted the generalizability of the study findings. Nonetheless, this may have been mitigated by the large sample size, high response rate, and the recruitment from different places across Palestine. Another limitation could be the small percentage of women aged 65 and older who represent the population with the highest risk for OC. It is noteworthy, however, that this age group comprises merely 5.8% of the total Palestinian population [44]. Including younger women, on the other hand, may facilitate implementation of future interventions that aim to increase awareness of this group.

## Conclusion

Only 5.1% of the study participants had good awareness of OC causation myths and were able to recognize 10 or more myths as incorrect. WBJ and the Gaza Strip participants were found to have similar likelihood to exhibit good awareness. The factors associated with low recognition of OC causation myths were earning higher monthly income as well as visiting hospitals and primary healthcare centers. Addressing mythical beliefs should be included in OC prevention strategies, and public health interventions in order to improve women’s understanding of OC risk factors and myths.

### Electronic supplementary material

Below is the link to the electronic supplementary material.

Supplementary Material 1

Supplementary Material 2

Supplementary Material 3

## Data Availability

The dataset used and analyzed during the current study will be available by the corresponding author upon reasonable request.

## Abbreviations

OC: ovarian cancer
WBJ: West Bank and Jerusalem
CI: confidence interval
OR: Odds ratio
