# Supplementary material for: Common misconceptions and myths about ovarian cancer causation: a national cross-sectional study from palestine
Source: BMC Public Health. 2024 Apr 12;24:1027. doi: 10.1186/s12889-024-18437-6 (PMC11015600; doi:10.1186/s12889-024-18437-6)
Supplement: Supplementary file 1 — Supplementary Material 1 [file 12889_2024_18437_MOESM1_ESM.docx]

# Common Misconceptions and Myths About Ovarian Cancer Causation: A National Cross-sectional Study from Palestine

Mohamedraed Elshami^1,2*^, Inas Jaber, MD^3*^, Mohammed Alser, MD ^4^, Ibrahim Al-Slaibi, MD^5^, Hadeel Jabr, MD^2^, Sara Ubaiat^6^, Aya Tuffaha, MD^7^, Salma Khader^3^, Reem Khraishi^8^, Zeina Abu Arafeh^3^, Sondos Al-Madhoun^9^, Aya Alqattaa^10^, Areej Yaseen^3^, Asmaa Abd El Hadi^10^, Ola Barhoush^3^, Maysun Hijazy^10^, Tamara Eleyan^3^, Amany Alser^9^, Amal Abu Hziema^10^, Amany Shatat^10^, Falasteen Almakhtoob^11^, Balqees Mohamad, MD^12^,Walaa Farhat^13^, Yasmeen Abuamra^14^, Hanaa Mousa^10^,Reem Adawi^3^, Alaa Musallam, MD^15^, Shurouq I. Albarqi, PharmB^16^, Nasser Abu-El-Noor, PhD^17#,^ and Bettina Bottcher, MD, PhD^10#^

*Contributed equally as a first co-author.

#Contributed equally as a senior co-author^.^

^1^Division of Surgical Oncology, University Hospitals Cleveland Medical Center, Cleveland, OH 44106

^2^Ministry of Health, Gaza, Palestine.

^3^Faculty of Medicine, Al-Quds University, Jerusalem, Palestine.

^4^ United Nations Relief and Works Agency for Palestine Refugees (UNRWA), Gaza, Palesine.

^5^Almakassed Hospital, Jerusalem, Palestine.

^6^Faculty of Medicine, Al-Quds University, Bethlehem, Palestine.

^7^Al-Watani Hospital, Nablus, Palestine

^8^Faculty of Medicine, An-Najah National University, Nablus, Palestine.

^9^Al-shiffa Hospital, Gaza, Palestine.

^10^ Faculty of Medicine, Islamic University of Gaza, Gaza, Palestine

^11^Facultyof Medicine, Palestine Polytechnic University, Hebron, Palestine.

^12^ Doctors Without Borders (Médecins Sans Frontières), Hebron, Palestine.

^13^Faculty of Medicine, Al-Quds University, Jenin, Palestine.

^14^ Faculty of Medicine, Al-Azhar University-Gaza, Gaza, Palestine.

^15^Al-Aqsa Hospital, Deir Albalah, Palestine.

^16^ Faculty of Pharmacy, Al-Azhar University of Gaza, Gaza, Palestine.

^17^Faculty of Nursing, Islamic University of Gaza, Gaza, Palestine

**Corresponding author**

Mohamedraed Elshami, MD, MMSc

Division of Surgical Oncology

Department of Surgery

University Hospitals Cleveland Medical Center

11100 Euclid Avenue, Lakeside 7100

Cleveland, OH 44106

Phone: 832-245-6055

Email: mohamedraed.elshami@gmail.com

Supplementary Table 1: Sensitivity analysis analyzing factors associated with having good recognition of the mythical causes of ovarian cancer.

| **Characteristic** | **Good recognition** | |
| --- | --- | --- |
|  | **AOR (95% CI)*** | **p-value** |
| **Age group**  18 to 44  45 or older | Ref  0.98 (0.68- 1.42) | Ref  0.93 |
| **Occupation**  Housewife  Employed  Retired  Student | Ref  0.87 (0.60- 1.25)  0.57 (0.08- 4.23)  1.31 (0.85- 2.01) | Ref  0.45  0.58  0.22 |
| **Monthly income**  < 1450 NIS  ≥ 1450 NIS | Ref  0.66 (0.51- 0.85) | Ref  0.002 |
| **Marital status**  Single  Married  Divorced/Widowed | Ref  0.69 (0.49- 1.02)  0.45 (0.19- 1.09) | Ref  0.06  0.08 |
| **Having a chronic disease**  No  Yes | Ref  0.69 (0.46- 1.03) | Ref  0.07 |
| **Site of data collection**  Public Spaces  Hospitals  Primary healthcare centers | Ref  0.65 (0.47- 0.90)  0.69 (0.51- 0.93) | Ref  0.009  0.014 |

AOR= adjusted odds ratio, CI= confidence interval.
* Adjusted for age-group, occupation, monthly income, marital status, having a chronic disease, and site of data collection.

Note: The outcome was dichotomized, where displaying good awareness was considered as ‘yes’, whereas displaying poor or fair awareness was considered as ‘no’.
