# Supplementary material for: Common misconceptions and myths about ovarian cancer causation: a national cross-sectional study from palestine
Source: BMC Public Health. 2024 Apr 12;24:1027. doi: 10.1186/s12889-024-18437-6 (PMC11015600; doi:10.1186/s12889-024-18437-6)
Supplement: Supplementary file 2 — Supplementary Material 2 [file 12889_2024_18437_MOESM2_ESM.docx]

# Common Misconceptions and Myths About Ovarian Cancer Causation: A National Cross-sectional Study from Palestine

Mohamedraed Elshami^1,2*^, Inas Jaber, MD^3*^, Mohammed Alser, MD ^4^, Ibrahim Al-Slaibi, MD^5^, Hadeel Jabr, MD^2^, Sara Ubaiat^6^, Aya Tuffaha, MD^7^, Salma Khader^3^, Reem Khraishi^8^, Zeina Abu Arafeh^3^, Sondos Al-Madhoun^9^, Aya Alqattaa^10^, Areej Yaseen^3^, Asmaa Abd El Hadi^10^, Ola Barhoush^3^, Maysun Hijazy^10^, Tamara Eleyan^3^, Amany Alser^9^, Amal Abu Hziema^10^, Amany Shatat^10^, Falasteen Almakhtoob^11^, Balqees Mohamad, MD^12^,Walaa Farhat^13^, Yasmeen Abuamra^14^, Hanaa Mousa^10^,Reem Adawi^3^, Alaa Musallam, MD^15^, Shurouq I. Albarqi, PharmB^16^, Nasser Abu-El-Noor, PhD^17#,^ and Bettina Bottcher, MD, PhD^10#^

*Contributed equally as a first co-author.

#Contributed equally as a senior co-author^.^

^1^Division of Surgical Oncology, University Hospitals Cleveland Medical Center, Cleveland, OH 44106

^2^Ministry of Health, Gaza, Palestine.

^3^Faculty of Medicine, Al-Quds University, Jerusalem, Palestine.

^4^ United Nations Relief and Works Agency for Palestine Refugees (UNRWA), Gaza, Palesine.

^5^Almakassed Hospital, Jerusalem, Palestine.

^6^Faculty of Medicine, Al-Quds University, Bethlehem, Palestine.

^7^Al-Watani Hospital, Nablus, Palestine

^8^Faculty of Medicine, An-Najah National University, Nablus, Palestine.

^9^Al-shiffa Hospital, Gaza, Palestine.

^10^ Faculty of Medicine, Islamic University of Gaza, Gaza, Palestine

^11^Facultyof Medicine, Palestine Polytechnic University, Hebron, Palestine.

^12^ Doctors Without Borders (Médecins Sans Frontières), Hebron, Palestine.

^13^Faculty of Medicine, Al-Quds University, Jenin, Palestine.

^14^ Faculty of Medicine, Al-Azhar University-Gaza, Gaza, Palestine.

^15^Al-Aqsa Hospital, Deir Albalah, Palestine.

^16^ Faculty of Pharmacy, Al-Azhar University of Gaza, Gaza, Palestine.

^17^Faculty of Nursing, Islamic University of Gaza, Gaza, Palestine

**Corresponding author**

Mohamedraed Elshami, MD, MMSc

Division of Surgical Oncology

Department of Surgery

University Hospitals Cleveland Medical Center

11100 Euclid Avenue, Lakeside 7100

Cleveland, OH 44106

Phone: 832-245-6055

Email: mohamedraed.elshami@gmail.com

Supplementary table 2: Multivariable logistic regression analyzing factors associated with the recognition of each mythical food-related cause of ovarian cancer.

AOR= adjusted odds ratio, CI= confidence interval, WBJ= West Bank and Jerusalem.
*Adjusted for age-group, educational level, occupation, monthly income, marital status, residency, having a chronic disease, knowing someone with cancer, and site of data collection.

| **Characteristic** | **Drinking from plastic bottles** | | **Eating burnt food** | | **Eating food containing artificial sweeteners** | | **Using microwave ovens** | |
| --- | --- | --- | --- | --- | --- | --- | --- | --- |
|  | **AOR (95% CI)*** | **p-value** | **AOR (95% CI)*** | **p-value** | **AOR (95% CI)*** | **p-value** | **AOR (95% CI)*** | **p-value** |
| **Age group**  18 to 44  45 or older | Ref  0.75 (0.63- 0.90) | Ref  0.002 | Ref  0.88 (0.74- 1.05) | Ref  0.17 | Ref  0.90 (0.74- 1.10) | Ref  0.31 | Ref  0.89 (0.72- 1.08) | Ref  0.24 |
| **Educational level**  Secondary or below  Post–secondary | Ref  0.95 (0.82- 1.10) | Ref  0.48 | Ref  1.05 (0.91- 1.22) | Ref  0.50 | Ref  0.98 (0.83- 1.15) | Ref  0.79 | Ref  0.99 (0.85- 1.17) | Ref  0.95 |
| **Occupation**  Unemployed/housewife  Employed  Retired  Student | Ref  0.92 (0.76- 1.11)  0.77 (0.34- 1.76)  1.45 (1.15- 1.83) | Ref  0.39  0.54  0.002 | Ref  0.90 (0.75- 1.09)  0.74 (0.35- 1.57)  1.36 (1.06- 1.73) | Ref  0.28  0.44  0.015 | Ref  0.88 (0.71- 1.08)  1.04 (0.47- 2.29)  1.17 (0.90- 1.52) | Ref  0.22  0.92  0.25 | Ref  1.05 (0.85- 1.29)  0.48 (0.17- 1.37)  1.12 (0.86- 1.46) | Ref  0.65  0.17  0.40 |
| **Monthly income**  < 1450 NIS  ≥ 1450 NIS | Ref  0.91 (0.75- 1.10) | Ref  0.32 | Ref  1.00 (0.82- 1.22) | Ref  0.97 | Ref  0.86 (0.70- 1.06) | Ref  0.16 | Ref  0.71 (0.57- 0.88) | Ref  0.002 |
| **Marital status**  Single  Married  Divorced/Widowed | Ref  0.82 (0.68- 0.98)  0.67 (0.45- 0.99) | Ref  0.028  0.044 | Ref  0.98 (0.81- 1.18)  1.16 (0.81- 1.67) | Ref  0.80  0.42 | Ref  0.81 (0.66- 0.99)  0.87 (0.58- 1.30) | Ref  0.043  0.49 | Ref  0.64 (0.53- 0.78)  0.49 (0.31- 0.76) | Ref  <0.001  0.002 |
| **Residency**  Gaza Strip  WBJ | Ref  0.99 (0.82- 1.20) | Ref  0.92 | Ref  1.97 (1.62- 2.41) | Ref  <0.001 | Ref  1.04 (0.85- 1.28) | Ref  0.72 | Ref  1.47 (1.18- 1.82) | Ref  0.001 |
| **Having a chronic disease**  No  Yes | Ref  0.85 (0.71- 1.02) | Ref  0.09 | Ref  0.89 (0.74- 1.06) | Ref  0.19 | Ref  1.00 (0.82- 1.21) | Ref  0.97 | Ref  0.94 (0.76- 1.15) | Ref  0.53 |
| **Knowing someone with cancer**  No  Yes | Ref  1.04 (0.92- 1.18) | Ref  0.55 | Ref  0.90 (0.79- 1.02) | Ref  0.11 | Ref  0.87 (0.76- 1.00) | Ref  0.048 | Ref  0.97 (0.84- 1.12) | Ref  0.71 |
| **Site of data collection**  Public Spaces  Hospitals  Primary healthcare centers | Ref  0.67 (0.57- 0.79)  0.74 (0.63- 0.86) | Ref  <0.001  <0.001 | Ref  1.04 (0.88- 1.23)  0.85 (0.73- 1.01) | Ref  0.63  0.06 | Ref  0.87 (.73- 1.04)  0.64 (0.54- 0.76) | Ref  0.13  <0.001 | Ref  0.88 (0.73- 1.06)  0.95 (0.80- 1.14) | Ref  0.19  0.58 |

| **Characteristic** | **Eating genetically modified food**  Supplementary table 2: Multivariable logistic regression analyzing factors associated with the recognition of each mythical food-related cause of ovarian cancer. (Ctd) | | | **Eating food containing additives** | |
| --- | --- | --- | --- | --- | --- |
|  | **AOR (95% CI)*** | **p-value** | **AOR (95% CI)*** | | **p-value** |
| **Age group**  18 to 44  45 or older | Ref  0.72 (0.57- 0.92) | Ref  0.007 | Ref  0.93 (0.72- 1.19) | | Ref  0.57 |
| **Educational level**  Secondary or below  Post–secondary | Ref  0.91 (0.76- 1.10) | Ref  0.34 | Ref  0.91 (0.75- 1.10) | | Ref  0.33 |
| **Occupation**  Unemployed/housewife  Employed  Retired  Student | Ref  0.83 (0.65- 1.07)  0.65 (0.20- 2.16)  0.94 (0.69- 1.29) | Ref  0.15  0.49  0.72 | Ref  0.99 (0.76- 1.28)  1.14 (0.43- 3.00)  1.26 (0.92- 1.72) | | Ref  0.92  0.80  0.15 |
| **Monthly income**  < 1450 NIS  ≥ 1450 NIS | Ref  0.60 (0.47- 0.77) | Ref  <0.001 | Ref  0.72 (0.55- 0.94) | | Ref  0.016 |
| **Marital status**  Single  Married  Divorced/Widowed | Ref  0.78 (0.62- 0.99)  0.94 (0.59- 1.50) | Ref  0.040  0.81 | Ref  0.76 (0.59- 0.96)  0.57 (0.32- 1.00) | | Ref  0.023  0.050 |
| **Residency**  Gaza Strip  WBJ | Ref  1.25 (0.97- 1.60) | Ref  0.08 | Ref  1.50 (1.15- 1.96) | | Ref  0.003 |
| **Having a chronic disease**  No  Yes | Ref  0.92 (0.73- 1.16) | Ref  0.48 | Ref  0.71 (0.54- 0.92) | | Ref  0.010 |
| **Knowing someone with cancer**  No  Yes | Ref  0.87 (0.74- 1.02) | Ref  0.08 | Ref  0.72 (0.61- 0.86) | | Ref  <0.001 |
| **Site of data collection**  Public Spaces  Hospitals  Primary healthcare centers  AOR= adjusted odds ratio, CI= confidence interval, WBJ= West Bank and Jerusalem. *Adjusted for age-group, educational level, occupation, monthly income, marital status, residency, having a chronic disease, knowing someone with cancer, and site of data collection. | Ref  0.94 (0.76- 1.16)  0.72 (0.59- 0.89) | Ref  0.56  0.002 | Ref  0.65 (0.51- 0.83)  1.05 (0.85- 1.30) | | Ref  <0.001  0.63 |
