# Supplementary material for: Common misconceptions and myths about ovarian cancer causation: a national cross-sectional study from palestine
Source: BMC Public Health. 2024 Apr 12;24:1027. doi: 10.1186/s12889-024-18437-6 (PMC11015600; doi:10.1186/s12889-024-18437-6)
Supplement: Supplementary file 3 — Supplementary Material 3 [file 12889_2024_18437_MOESM3_ESM.docx]

# Common Misconceptions and Myths About Ovarian Cancer Causation: A National Cross-sectional Study from Palestine

Mohamedraed Elshami^1,2*^, Inas Jaber, MD^3*^, Mohammed Alser, MD ^4^, Ibrahim Al-Slaibi, MD^5^, Hadeel Jabr, MD^2^, Sara Ubaiat^6^, Aya Tuffaha, MD^7^, Salma Khader^3^, Reem Khraishi^8^, Zeina Abu Arafeh^3^, Sondos Al-Madhoun^9^, Aya Alqattaa^10^, Areej Yaseen^3^, Asmaa Abd El Hadi^10^, Ola Barhoush^3^, Maysun Hijazy^10^, Tamara Eleyan^3^, Amany Alser^9^, Amal Abu Hziema^10^, Amany Shatat^10^, Falasteen Almakhtoob^11^, Balqees Mohamad, MD^12^,Walaa Farhat^13^, Yasmeen Abuamra^14^, Hanaa Mousa^10^,Reem Adawi^3^, Alaa Musallam, MD^15^, Shurouq I. Albarqi, PharmB^16^, Nasser Abu-El-Noor, PhD^17#,^ and Bettina Bottcher, MD, PhD^10#^

*Contributed equally as a first co-author.

#Contributed equally as a senior co-author^.^

^1^Division of Surgical Oncology, University Hospitals Cleveland Medical Center, Cleveland, OH 44106

^2^Ministry of Health, Gaza, Palestine.

^3^Faculty of Medicine, Al-Quds University, Jerusalem, Palestine.

^4^ United Nations Relief and Works Agency for Palestine Refugees (UNRWA), Gaza, Palesine.

^5^Almakassed Hospital, Jerusalem, Palestine.

^6^Faculty of Medicine, Al-Quds University, Bethlehem, Palestine.

^7^Al-Watani Hospital, Nablus, Palestine

^8^Faculty of Medicine, An-Najah National University, Nablus, Palestine.

^9^Al-shiffa Hospital, Gaza, Palestine.

^10^ Faculty of Medicine, Islamic University of Gaza, Gaza, Palestine

^11^Facultyof Medicine, Palestine Polytechnic University, Hebron, Palestine.

^12^ Doctors Without Borders (Médecins Sans Frontières), Hebron, Palestine.

^13^Faculty of Medicine, Al-Quds University, Jenin, Palestine.

^14^ Faculty of Medicine, Al-Azhar University-Gaza, Gaza, Palestine.

^15^Al-Aqsa Hospital, Deir Albalah, Palestine.

^16^ Faculty of Pharmacy, Al-Azhar University of Gaza, Gaza, Palestine.

^17^Faculty of Nursing, Islamic University of Gaza, Gaza, Palestine

**Corresponding author**

Mohamedraed Elshami, MD, MMSc

Division of Surgical Oncology

Department of Surgery

University Hospitals Cleveland Medical Center

11100 Euclid Avenue, Lakeside 7100

Cleveland, OH 44106

Phone: 832-245-6055

Email: mohamedraed.elshami@gmail.com

AOR= adjusted odds ratio, CI= confidence interval, WBJ= West Bank and Jerusalem.
*Adjusted for age-group, educational level, occupation, monthly income, marital status, residency, having a chronic disease, knowing someone with cancer, and site of data collection.

Supplementary table 3: Multivariable logistic regression analyzing factors associated with the recognition of each other mythical cause of ovarian cancer.

AOR= adjusted odds ratio, CI= confidence interval, WBJ= West Bank and Jerusalem.
*Adjusted for age-group, educational level, occupation, monthly income, marital status, residency, having a chronic disease, knowing someone with cancer, and site of data collection.

| **Characteristic** | **Having a physical trauma** | | **Using aerosol containers** | | **Using cleaning products** | | **Feeling stressed** | |
| --- | --- | --- | --- | --- | --- | --- | --- | --- |
|  | **AOR (95% CI)*** | **p-value** | **AOR (95% CI)*** | **p-value** | **AOR (95% CI)*** | **p-value** | **AOR (95% CI)*** | **p-value** |
| **Age group**  18 to 44  45 or older | Ref  0.91 (0.78- 1.05) | Ref  0.20 | Ref  0.72 (0.62- 0.84) | Ref  <0.001 | Ref  0.79 (0.67- 0.92) | Ref  0.003 | Ref  0.78 (0.66- 0.92) | Ref  0.003 |
| **Educational level**  Secondary or below  Post–secondary | Ref  1.10 (0.97- 1.25) | Ref  0.14 | Ref  1.06 (0.93- 1.20) | Ref  0.36 | Ref  0.85 (0.74- 0.97) | Ref  0.014 | Ref  0.89 (0.77- 1.02) | Ref  0.09 |
| **Occupation**  Unemployed/housewife  Employed  Retired  Student | Ref  0.87 (0.74- 1.02)  0.88 (0.48- 1.60)  1.03 (0.83- 1.29) | Ref  0.09  0.67  0.79 | Ref  0.79 (0.67- 0.93)  0.85 (0.45- 1.61)  1.05 (0.85- 1.32) | Ref  0.005  0.62  0.64 | Ref  0.90 (0.76- 1.07)  0.64 (0.30- 1.34)  1.32 (1.05- 1.66) | Ref  0.25  0.24  0.017 | Ref  0.98 (0.82- 1.17)  1.01 (0.50- 2.03)  0.96 (0.76- 1.22) | Ref  0.79  0.98  0.74 |
| **Monthly income**  < 1450 NIS  ≥ 1450 NIS | Ref  1.11 (0.94- 1.30) | Ref  0.23 | Ref  1.04 (0.88- 1.23) | Ref  0.62 | Ref  0.96 (0.80- 1.14) | Ref  0.62 | Ref  1.07 (0.90- 1.28) | Ref  0.46 |
| **Marital status**  Single  Married  Divorced/Widowed | Ref  1.28 (1.08- 1.50)  1.13 (0.83- 1.55) | Ref  0.003  0.44 | Ref  0.86 (0.73- 1.01)  0.85 (0.61- 1.18) | Ref  0.07  0.33 | Ref  0.92 (0.77- 1.10)  0.93 (0.66- 1.31) | Ref  0.36  0.70 | Ref  0.90 (0.76- 1.06)  0.67 (0.46- 0.98) | Ref  0.26  0.038 |
| **Residency**  Gaza Strip  WBJ | Ref  0.74 (0.63- 0.86) | Ref  <0.001 | Ref  1.28 (1.08- 1.51) | Ref  0.004 | Ref  1.01 (0.85- 1.20) | Ref  0.91 | Ref  0.91 (0.76- 1.09) | Ref  0.29 |
| **Having a chronic disease**  No  Yes | Ref  0.92 (0.79- 1.07) | Ref  0.29 | Ref  0.89 (0.76- 1.04) | Ref  0.14 | Ref  0.99 (0.84- 1.16) | Ref  0.90 | Ref  0.93 (0.79- 1.10) | Ref  0.40 |
| **Knowing someone with cancer**  No  Yes | Ref  1.33 (1.19- 1.49) | Ref  <0.001 | Ref  1.03 (0.92- 1.15) | Ref  0.61 | Ref  0.94 (0.84- 1.06) | Ref  0.31 | Ref  0.96 (0.85- 1.08) | Ref  0.52 |
| **Site of data collection**  Public Spaces  Hospitals  Primary healthcare centers | Ref  0.88 (0.77- 1.02)  0.79 (0.69- 0.90) | Ref  0.09  0.001 | Ref  0.94 (0.81- 1.08)  0.87 (0.75- 1.00) | Ref  0.38  0.050 | Ref  1.05 (0.90- 1.23)  0.99 (0.85- 1.15) | Ref  0.51  0.88 | Ref  1.08 (0.93- 1.26)  0.77 (0.66- 0.90) | Ref  0.31  0.001 |

Supplementary table 3: Multivariable logistic regression analyzing factors associated with the recognition of each other mythical cause of ovarian cancer. (Ctd)

| **Characteristic** | **Living near power lines** | | **Exposure to electromagnetic frequencies** | | **Using mobile phones** | |
| --- | --- | --- | --- | --- | --- | --- |
|  | **AOR (95% CI)*** | **p-value** | **AOR (95% CI)*** | **p-value** | **AOR (95% CI)*** | **p-value** |
| **Age group**  18 to 44  45 or older | Ref  0.76 (0.63- 0.91) | Ref  0.002 | Ref  1.03 (0.87- 1.22) | Ref  0.75 | Ref  0.70 (0.58- 0.84) | Ref  <0.001 |
| **Educational level**  Secondary or below  Post–secondary | Ref  0.91 (0.79- 1.05) | Ref  0.20 | Ref  0.87 (0.75- 1.01) | Ref  0.06 | Ref  1.16 (1.00- 1.33) | Ref  0.047 |
| **Occupation**  Unemployed/housewife  Employed  Retired  Student | Ref  0.86 (0.71- 1.04)  0.68 (0.28- 1.65)  1.16 (0.91- 1.48) | Ref  0.12  0.40  0.24 | Ref  0.96 (0.80- 1.16)  0.64 (0.29- 1.40)  0.92 (0.72- 1.18) | Ref  0.66  0.26  0.52 | Ref  0.87 (0.72- 1.04)  1.26 (0.64- 2.50)  1.09 (0.86- 1.38) | Ref  0.13  0.51  0.46 |
| **Monthly income**  < 1450 NIS  ≥ 1450 NIS | Ref  0.94 (0.78- 1.13) | Ref  0.51 | Ref  0.83 (0.69- 1.00) | Ref  0.051 | Ref  1.03 (0.85- 1.25) | Ref  0.74 |
| **Marital status**  Single  Married  Divorced/Widowed | Ref  0.89 (0.74- 1.10)  0.71 (0.48- 1.05) | Ref  0.23  0.09 | Ref  0.77 (0.64- 0.93)  0.61 (0.42- 0.89) | Ref  0.007  0.011 | Ref  0.68 (0.56- 0.81)  0.57 (0.39- 0.85) | Ref  <0.0010.006 |
| **Residency**  Gaza Strip  WBJ | Ref  0.54 (0.45- 0.65) | Ref  <0.001 | Ref  1.03 (0.86- 1.24) | Ref  0.73 | Ref  1.23 (1.02- 1.49) | Ref  0.032 |
| **Having a chronic disease**  No  Yes | Ref  0.95 (0.79- 1.14) | Ref  0.57 | Ref  1.05 (0.88- 1.25) | Ref  0.58 | Ref  1.01 (0.84- 1.21) | Ref  0.95 |
| **Knowing someone with cancer**  No  Yes | Ref  1.21 (1.07- 1.38) | Ref  0.003 | Ref  0.99 (0.88- 1.12) | Ref  0.89 | Ref  0.99 (0.88- 1.13) | Ref  0.93 |
| **Site of data collection**  Public Spaces  Hospitals  Primary healthcare centers  AOR= adjusted odds ratio, CI= confidence interval, WBJ= West Bank and Jerusalem. *Adjusted for age-group, educational level, occupation, monthly income, marital status, residency, having a chronic disease, knowing someone with cancer, and site of data collection. | Ref  0.78 (0.66- 0.91)  0.64 (0.55- 0.75) | Ref  0.002  <0.001 | Ref  1.25 (1.07- 1.47)  0.80 (0.68- 0.94) | Ref  0.006  0.006 | Ref  0.71 (0.61- 0.84)  0.70 (0.60- 0.82) | Ref  <0.001<0.001 |
